# Quality of life in patients with gastric cancer: translation and psychometric evaluation of the Iranian version of EORTC QLQ-STO22

**DOI:** 10.1186/1471-2407-9-305

**Published:** 2009-08-28

**Authors:** Sanamber Sadighi, Ali Montazeri, Zahra Sedighi, Mohammad Ali Mohagheghi, Hossein Froutan

**Affiliations:** 1 Cancer Research Center, Cancer Institute, Tehran University of Medical Sciences, Tehran, Iran; 2 Department of Mental Health, Iranian Institute for Health Sciences Research, ACECR, Tehran, Iran; 3 Public Health and Health Policy, Division of Community-Based Sciences, Faculty of Medicine, University of Glasgow, Glasgow, UK; 4 Department of Gastroentrology, Tehran University of Medical Sciences, Tehran, Iran

## Abstract

**Background:**

Disease and treatment related events, can adversely affect the quality of life of patients with cancer. The purpose of this study was to translate and validate a gastric cancer specific health related quality of life questionnaire (EORTC QLQ-STO22) for Iranian patients suffering from gastric cancer.

**Methods:**

Forward-backward procedure was applied to translate the English language version of the EORTC QLQ-STO22 into Persian (Iranian language). Then, the questionnaire and the EORTC core quality of life instrument (QLQ-C30) were administered to a sample of patients with confirmed diagnosis of gastric cancer. All patients filled in questionnaires before and after one month of treatment. Patients were divided into two groups based on intension of treatment (curative vs. palliative). Reliability and validity of the module was tested by internal consistency and known group comparisons, respectively.

**Results:**

In all, 105 patients were entered into the study. Cronbach's alpha for multi-item scales (to test reliability) ranged from 0.54 to 0.87. The questionnaire discriminated well between clinically distinct subgroups of patients both before and after treatment lending support to its convergent and clinical validity.

**Conclusion:**

Overall, the Iranian version of the EORTC QLQ-STO22 demonstrated a good reliability and clinical validity to support its use in combination with core questionnaire in outcome studies of gastric cancer in Iran. However, using the QLQ-STO22 in a wide range of Iranian patients with gastric cancer should allow further confirmation for its psychometric properties.

## Background

Although mortality rates due to gastric cancer had been declining for several decades, on a worldwide scale its incidence is still high, and it is the second leading cause of cancer death, behind lung cancer [1,2]. Recent efforts to improve survival include pre and post-operative chemotherapy and chemo-radiotherapy. However, improvements in survival with multi-modal treatment may also be associated with increased toxic side effects. Therefore full evaluation of new treatments of gastric cancer should be included patient-reported outcome measures such as health related quality of life (HRQOL) as well as assessment of biomarkers, pathologic responses or survival outcomes.

Although quality of life in patients with gastric cancer is increasingly added as outcome measure in clinical research, it is argued that quality of life assessments in these patients deserve more systematic studies using gastric cancer specific instruments. A recent review of the literature on quality of life in gastric cancer indicated that in most reported studies quality of life was assessed mainly with generic measures, and the social dimensions of quality of life were largely neglected [3]. In assessing quality of life in cancer patients it is recommended that to use a cancer-specific questionnaire as a general measure of quality of life in cancer patients (e.g. the EORTC QLQ-C30) plus site-specific modules (e.g. breast cancer specific or gastric cancer specific). Thus, for instance the EORTC, in addition to core cancer quality of life questionnaire, has developed several site-specific questionnaires including gastric specific quality of life measures (the EORTC QLQ-STO22) in order to collect more relevant patient-reported outcomes in studying quality of life in this group of cancer patients.

The QLQ-STO22 has been translated into many languages such Dutch, Danish, French, German, Geek, Hungarian, Italian, Japanese, Korea, Norwegian, Portuguese, Brazilian, Russian, Spanish, Taiwanese and Turkish [4,5].

Gastric cancer is the most common cancer in Iran and according to the latest published data there are more than 5000 new cases and equally about 5000 deaths each year due to gastric cancer [6]. Thus as one might realize studying quality of life in gastric cancer patients in Iran is very important and relevant. Since the EORTC QLQ-STO22 was not available in Iran, this study carried out to translate and provide evidence for its psychometric properties in Iran so that the questionnaire could be used in the future outcome studies in gastric cancer patients with the hope that this might contribute to the existing literature and help to improve quality of life among these cancer patients.

## Methods

### Design and data collection

This was an observational 4-week follow up study conducted in the Medical Oncology Department of the Cancer Research Center of the Tehran University of Medical Science. A consecutive sample of patients were entered into the study during March 2005 to September 2007. Eligible cases were gastric cancer patients with confirmed diagnosis and life expectancy of at least 4 weeks. Patients were excluded if they had concurrent malignancies or if they were unable to understand the questionnaire.

### The questionnaire

Permission was asked from the EORTC Quality of Life Department to develop the Iranian version of the EORTC gastric cancer specific quality of life questionnaire (EORTC QLQ-STO22). We used the standard 'forward-backward' procedure in order to translate the English language version of the EORTC QLQ-STO22 into Persian (Iranian language). The translated module was reviewed, pre-tested, revised and its final form was used in this study. In addition the Iranian version of the EORTC QLQ-C30 was administered to patients. The psychometric properties of the Iranian version of the EORTC QLQ-C30 are well documented [7]. Patients completed the questionnaires before starting chemotherapy or supportive care. The second assessment was carried out four weeks later. At baseline assessment, patients were asked to complete a short debriefing questionnaire about the time took to complete the questionnaires, the need for help in completing the questionnaire and indicating if any of the items appeared confusing, difficult to answer or upsetting. Demographic and treatment data were also recorded.

The EORTC QLQ-STO22 module contains 22 items in a similar layout and response format to the EORTC QLQ-C30. The module consists of five multi-item scales (dysphagia, eating restrictions, pain, reflux, and anxiety) and four single items (dry mouth, body image, hair loss, and taste problem). Higher scores on the QLQ-STO22 represent greater level of symptoms [5].

### Statistical analysis

Reliability: internal consistency and test-retest analyses were performed to test reliability. The internal consistency of the multi-item scales was assessed by Cronbach's alpha coefficient at baseline and four week later. Values equal to or greater than 0.7 were considered satisfactory [8]. Test-retest reliability of the questionnaire was examined using intraclass correlation coefficients (ICC) between pre- and post-treatment assessments. Values of ICC vary from zero (totally unreliable) to 1 (perfectly reliable). Values above 0.80 were considered as evidence of excellent reliability [9].

Validity: convergent validity and clinical validity were performed to examine scale validity. Convergent validity for each scale was assessed using the correlation between each item and its own scale corrected for overlap. It was expected that the correlation between an item and its own scale was significantly higher than its correlation with other scales. Pearson's correlation coefficient was used to test convergent validity and values of 0.40 or above were considered satisfactory (r ≥ 0.81-1.0 as excellent, 0.61-0.80 very good, 0.41-0.60 good, 0.21-0.40 fair, and 0-0.20 poor) [8,9].

Known group comparisons was used for analysis of the clinical validity of the Iranian version of the QLQ-STO22 in order to explore the extent to which the questionnaire is able to discriminate between subgroups of patients. Known groups used for this comparison were treatment groups (potentially curative vs. palliative). Group differences were assessed using a non-parametric test (Mann-Whitney U test).

### Ethics

Ethics committee of Tehran University of Medical Sciences approved the study. Written informed consent was obtained from all enrolled patients.

## Results

In total, 105 patients filled in both questionnaires the EORTC QLQ-C30 and QLQ-STO22. Of these, 50 had loco-regional disease and received curative therapy (chemotherapy and surgery), while 55 had advanced disease and poor performance and received palliative treatment. The mean age of patients was 58.1 years (SD = 10.7), and 76% were male (n = 72). The average time required to complete the questionnaire was 5 minutes. Almost all patients found the questionnaire easy to understand and acceptable. However, a few patients commented that they could not differentiate between 'acid indigestion and hurt burn' (item 39) or some patients stated that they could not understand what does 'trouble with belching' really mean while in general belching is unpleasant condition (item 40).

Table 1 shows the internal consistency for the five QLQ-STO22 multi-item scales. In general all scales except eating restrictions (α = 0.54) showed satisfactory results. Intraclass correlation coefficient (ICC) values for the Iranian version of QLQ-STO22 also indicated acceptable test-retest reliability for the questionnaire. ICC values ranged from 0.53 for eating restrictions to 0.84 for dysphagia.

**Table 1:** Descriptive statistics and scale reliability of the EORTC QLQ-STO22 (n = 105)

	Pre treatment		Follow up	
	**Mean (SD)**	Cronhbach's alpha*	**Mean (SD)**	Cronhbac's alpha*

Multi-item Scales**				

Dysphagia	21.8 (25.0)	0.72	20.0 (26.3)	0.81

Stomach pain	33.1 (26.3)	0.78	30.1 (26.3)	0.87

Reflux	26.8 (23.1)	0.62	27.2 (24.5)	0.74

Eating restrictions	32.8 (14.0)	0.54	31.1 (15.2)	0.61

Anxiety	56 (31.3)	0.87	55.8 (32.8)	0.93

* A value of 0.70 or above indicates adequate reliability

** Scores range from 0 to 100 (higher scores indicate worse conditions)

Item convergent validity of the QLQ-STO22 is shown in Table 2. There were a desirable correlation between each item and its own scale lending support to its item-component validity. As indicated in Table 2 the correlation between an item and its own scale was significantly higher than its correlation with other scales.

**Table 2:** Correlation between multi-item scales and its own scale corrected for overlap (figures are Pearson correlation coefficient obtained from pretreatment assessment)*

	Dysphagia	Pain	Reflux	Eating restrictions	Anxiety
**Dysphagia**					

Problems eating solid foods	**0.82**	0.52	0.40	0.22	0.21

Problems eating liquidized or soft food	**0.87**	0.34	0.29	0.01	0.08

Problems drinking liquids	**0.78**	0.17	0.23	0.04	0.03

**Pain**					

Eating discomfort	0.61	**0.76**	0.55	0.32	0.33

Stomach pain	0.31	**0.84**	0.38	0.34	0.23

Stomach discomfort	0.27	**0.89**	0.46	0.42	0.31

Abdomen bolting	0.21	**0.62**	0.41	0.24	0.36

**Reflux**					

Acid or bile problem	0.30	0.44	**0.83**	0.21	0.30

Acid indigestion or heartburn	0.24	0.52	**0.73**	0.21	0.18

Belching trouble	0.35	0.33	**0.69**	0.39	0.25

**Eating restrictions**					

Full up too quickly	0.34	0.54	0.46	**0.74**	0.35

Trouble enjoying meals	0.38	0.34	0.20	**0.72**	0.08

Long time to complete meals	0.20	0.40	0.33	**0.74**	0.38

Eating trouble in front of other people	0.08	0.46	0.35	**0.77**	0.20

**Anxiety**					

Thinking about illness	0.19	0.39	0.31	0.43	**0.92**

Worry about low weight	0.18	0.42	0.37	0.34	**0.85**

Health worry	0.04	0.28	0.01	0.07	**0.46**

* Similar results were obtained from the post-treatment assessment. To avoid confusion the correlation coefficients are not presented in this table.

** Excellent correlation ≥ 0.81-1.0, 0.61-0.80 very good, 0.41-0.60 good, 0.21-0.40 fair, and 0-0.20 poor

Table 3 and Table 4 are presenting the results obtained from known group comparisons before and after treatment. In both assessments, patients in different groups that are curative and palliative treatment groups showed significant differences for most quality of life scores.

**Table 3:** The known groups comparison before treatment

	Curative treatment (n = 50)	Palliative treatment (n = 55)	
	**Mean (SD)**	**Mean (SD)**	P*

**EORTC QLQ-C30 **			

Functioning scores**			

Physical functioning	75.3 (15.8)	55.1 (23.5)	< 0.0001

Role functioning	(19.5) 77.7	60.3 (27.3)	0.001

Emotional functioning	67.1 (25.1)	53.4 (26.6)	0.008

Cognitive functioning	96.4 (10.9)	90.6 (14.7)	0.009

Social functioning	78.6 (16.7)	(25.2)71.8	0.26

Global quality of life	45.7 (11.8)	39.2 (10.5)	0.007

Symptom scores***			

Fatigue	36.0 (24.4)	56.8 (27.1)	< 0.0001

Nausea & vomiting	10.0 (22.3)	24.2 (28.1)	0.001

Pain	30.4 (24.1)	52.8 (28.3)	< 0.0001

Dyspnea	6.0 (14.5)	9.6 (20.9)	0.51

Insomnia	27.3 (33.4)	46.0 (35.4)	0.006

Appetite loss	34.0 (38.9)	54.0 (37.6)	0.007

Constipation	10.6 (24.6)	27.7 (37.6)	0.009

Diahrea	5.3 (15.5)	5.5 (16.8)	0.91

Financial difficulties	80.8 (30.0)	72.8 (36.6)	0.26

QLQ-STO22 scores***			

Dysphagia	15.6 (20.6)	29.1 (28.3)	0.01

Stomach area pain	25.0 (24.4)	38.3 (24.4)	0.005

Reflux	20.3 (20.1)	30.3 (24.6)	0.03

Eating restrictions	29.9 (14.9)	33.3 (16.1)	0.44

Anxiety	50.3 (27.6)	65.3 (29.4)	0.01

Dry mouth	29.2 (35.1)	52.3 (35.3)	0.001

Taste problem	20.4 (32.5)	31.2 (33.6)	0.06

Body image	20.9 (24.7)	26.6 (33.5)	0.73

Hair loss	17.3 (33.4)	18.8 (27.2)	0.51

* P values derived from Mann-Whitney test

** Scores range from 0 to 100 with higher scores indicating better conditions.

*** Scores range from 0 to 100 with higher scores indicating greater level of symptoms.

**Table 4:** The know groups comparison after treatment

	Curative treatment (n = 50)	Palliative treatment (n = 55)	
	**Mean (SD)**	**Mean (SD)**	P*

**EORTC QLQ-C30**			

Functioning scores**			

Physical functioning	71.4 (21.8)	52.7 (23.6)	< 0.0001

Role functioning	74.4 (22.6)	61.2 (27.4)	0.02

Emotional functioning	66.2 (26.1)	56.9 (25.5)	0.08

Cognitive functioning	94.4 (12.8)	89.1 (17.7)	0.07

Social functioning	79.4 (16.1)	68.9 (26.8)	0.12

Global quality of life	50.1 (18.2)	38.4 (11.9)	0.003

Symptom scores***			

Fatigue	40.0 (26.1)	54.3 (29.9)	0.01

Nausea & vomiting	15.9 (19.8)	33.3 (32.7)	0.01

Pain	25.9 (22.0)	43.3 (28.0)	0.001

Dyspnea	2.8 (9.4)	12.0 (20.1)	0.009

Insomnia	29.7 (31.6)	42.7 (35.5)	0.07

Appetite loss	10.6 (24.6)	27.7 (37.6)	0.009

Constipation	10.8 (23.3)	19.1 (27.5)	0.05

Diahrea	11.5 (21.3)	15.6 (28.5)	0.63

Financial difficulties	78.0 (32.1)	77.7 (33.3)	0.89

QLQ-STO22 scores***			

Dysphagia	14.0 (23.0)	27.2 (28.6)	0.009

Stomach area pain	18.1 (19.3)	43.6 (26.3)	< 0.0001

Reflux	20.2 (21.8)	36.3 (24.5)	0.001

Eating restrictions	29.9 (15.1)	34.3 (15.4)	0.09

Anxiety	46.0 (34.7)	58.8 (32.3)	0.15

Dry mouth	38.5 (36.8)	53.6 (36.4)	0.05

Taste problem	24.0 (30.2)	35.8 (34.9)	0.11

Body image	25.0 (35.7)	38.3 (37.8)	0.25

Hair loss	39.6 (33.1)	51.4 (34.6)	0.14

* P values derived from Mann-Whitney test

** Scores range from 0 to 100 with higher scores indicating better conditions.

*** Scores range from 0 to 100 with higher scores indicating greater level of symptoms.

## Discussion

This was a validation study of the EORTC QLO-STO22 in Iran and in general the questionnaire showed promising psychometric results. In addition patients received it well and we did not notice any problems when it was administered to the Iranian patients reflecting the fact that the translation was satisfactory and easy to understand.

In general the reliability of the Iranian version of the QLQ-STO22 was relatively good. However, internal consistency for two multi-item scales (reflux and eating restrictions) was lower than recommended value. The internal consistency for reflux subscale at baseline assessment was 0.62 and for eating restrictions at both assessments was 0.54, and 0.61 respectively. It seems that since the internal consistency for the reflux improved in the second assessment thus it could be regarded as satisfactory. But it appears that the low internal consistency for the eating restrictions subscale could not be neglected. It seems that item 42 (have you had trouble enjoying your meals?) that belongs to this subscale needs some modification in the future studies. Item 42 is a very straightforward question and we did not have nor any problems in translating the item into Persian language nor any patients indicated difficulties in responding to this question. Yet, we are not certain why the internal consistency for this scale was lower than recommended value. Perhaps 'enjoyment of meals' might mean different thing in our culture as compared to the meaning of 'enjoyment of meals' in western countries. It is not surprising that a similar study from Taiwan also reported that the internal consistency of the eating restrictions subscale was lower that recommended value (0.67) [10]. In addition, although a study from Japan reported high internal consistency for all five multi-item subscales including eating restrictions subscale (ranging from 0.76 to 0.88), it was found that item 42 showed higher correlation with dysphagia scale and even in factor analysis loaded highly on pain subscale rather that eating restrictions subscale. As suggested there might be a need to discuss with EORTC Quality of Life Group to establish an agreement on the various language versions of the QLQ-STO22 [11].

Clinical validity of the questionnaire, as examined by using known-group comparisons, showed satisfactory results. The questionnaire discriminated well between two groups that differed in their clinical status receiving two different regimens. Differences in quality of life scores on both measures (QLQ-C30 and QLQ-STO22) between patients who received curative treatment and those who received palliative treatment in most instances were significant at pre- and post-treatment assessments (Table 3 and Table 4). However, unlike most studies already cited in this paper there were no significant differences between two groups for social functioning. One possible explanation for such observation might be related to the fact that in Iran social ties are relatively very strong and thus both patient groups received equal support from family, relatives and friends.

The present study indicated that global quality of life was the most adversely affected subscale among the respondents while others have shown that patients scored lower on social functioning [12]. Lower score for global quality of life in other cancer patients were also reported. For instance, a recent study form Kuwait (very similar in culture to our patients) reported that global quality of life in breast cancer was lower than other functioning scores [13]. Such similarities or dissimilarities between patients from different cultures might worth to be studied further. However, it seems that effective treatment could help to improve quality of life in gastric cancer patients [14,15].

Although there was excellent patient compliance, we need more exploration of cross-cultural differences. Further studies with larger samples are also needed to confirm the sensitivity to changes over time.

## Conclusion

Overall the Iranian version of the EORTC QLQ-STO22 showed that it is a reliable and valid specific measure of quality of life in patients with gastric cancer. However, using the QLQ-STO22 in a wide range of Iranian patients with gastric cancer should allow further confirmation for its psychometric properties.

## Competing interests

The authors declare that they have no competing interests.

## Authors' contributions

SS and AM were the main investigators. SS wrote the first draft and designed the study. AM analyzed the data and wrote the final manuscript. ZS contributed to the data collection and data entry. MAM and HF contributed to the study design and patient management. All authors read and approved the final manuscript.

## Pre-publication history

The pre-publication history for this paper can be accessed here:

http://www.biomedcentral.com/1471-2407/9/305/prepub
